# Performance of a Double RIS Communication System Aided by Partially Active Elements

**DOI:** 10.3390/s23146338

**Published:** 2023-07-12

**Authors:** Seung-Geun Yoo, Min-A Kim, Jin-Woo Kim, Sang-Wook Park, Young-Hwan You, Hyoung-Kyu Song

**Affiliations:** 1 Department of Information and Communication Engineering, Sejong University, Seoul 05006, Republic of Korea; dbtmdrms96@naver.com (S.-G.Y.); happy990927@naver.com (M.-A.K.); kjwccm@naver.com (J.-W.K.); share1211@naver.com (S.-W.P.); 2 Department of Convergence Engineering for Intelligent Drone, Sejong University, Seoul 05006, Republic of Korea; yhyou@sejong.ac.kr; 3 Department of Computer Engineering, Sejong University, Seoul 05006, Republic of Korea

**Keywords:** reconfigurable intelligent surface (RIS), double RIS, active RIS, singular value decomposition (SVD)

## Abstract

Reconfigurable intelligent surface (RIS) has emerged as a promising technology to enhance the spectral efficiency of wireless communication systems. However, if there are many obstacles between the RIS and users, a single RIS may not provide sufficient performance. For this reason, a double RIS-aided communication system is proposed in this paper. However, this system also has a problem: the signal is attenuated three times due to the three channels created by the double RIS. To overcome these attenuations, an active RIS is proposed in this paper. An active RIS is almost the same as a conventional RIS, except for the included amplifier. Comprehensively, the proposed system overcomes various obstacles and attenuations. In this paper, an active RIS is applied to the second RIS. To reduce the power consumption of active elements, a partially active RIS is applied. To optimize the RIS elements, the sum of the covariance matrix is found by using channels related to each RIS, and the right singular vector is exploited using singular value decomposition for the sum of the covariance matrix. Then, the singular value of the sum of the covariance value is checked to determine which element is the active element. Simulation results show that the proposed system has better sum rate performance compared to a single RIS system. Although it has a lower sum rate performance compared to a double RIS with fully active elements, the proposed system will be more attractive in the future because it has much better energy efficiency.

## 1. Introduction

As communication users want higher data rates and lower latency, the frequency band used in communication systems has been expanding. However, utilizing higher frequency bands, such as millimeter wave (mmWave) [1,2], presents significant problems. As the frequency increases, the channel of the communication system experiences high attenuation. Thus, communication coverage and performance are limited.

Reconfigurable intelligent surface (RIS) has emerged as a promising technology to deal with problems and to improve the spectral efficiency of wireless communication systems [3,4,5] by creating a reflected link. The RIS is a rectangular surface with a large number of nearly passive elements. The phase and amplitude of each element can be dynamically adjusted. A conventional communication system has limited performance when the direct link between the base station (BS) and users is blocked by obstacles such as buildings and walls. However, since a single RIS-aided communication system has two links, this system can overcome environmental limitations and achieve better performance. The two links are as follows: a direct link and the other is reflected through the RIS. Structurally, a single RIS-aided communication system is nearly the same, with cooperative communication, such as decode and forward and amplify and forward. However, there are some differences [6,7,8,9]. For example, cooperative communication requires active electronic components like digital-to-analog and analog-to-digital, but a single RIS-aided communication system does not need active electronic components, resulting in less energy consumption [6].

**Figure 1:**
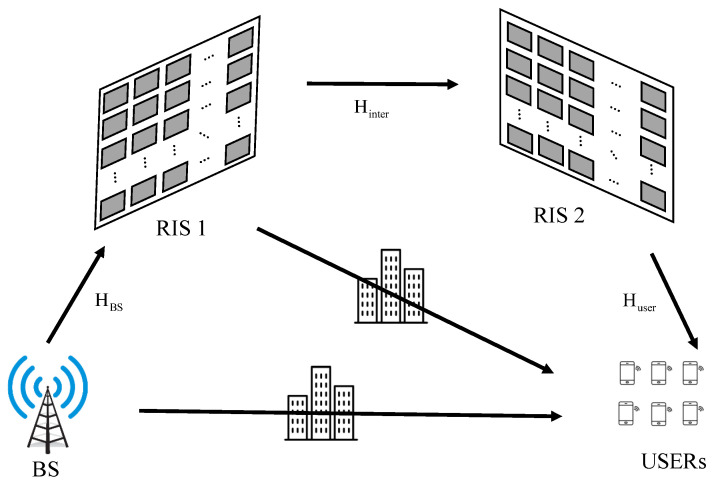
Double RIS MISO communication system.

**Figure 2:**
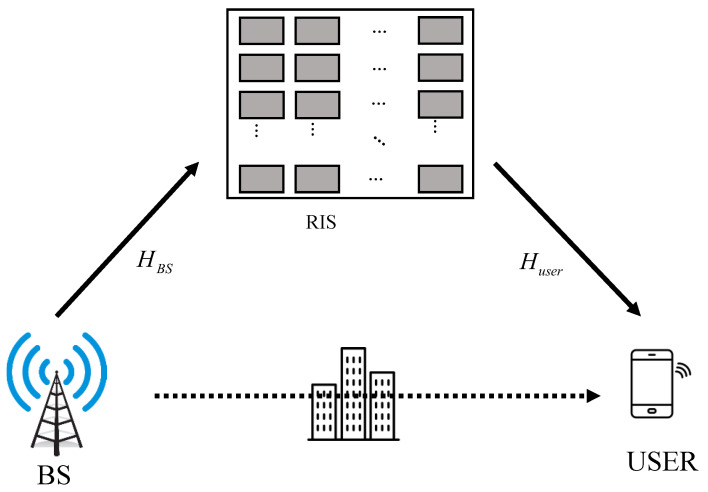
Single RIS MISO communication system.

**Figure 3:**
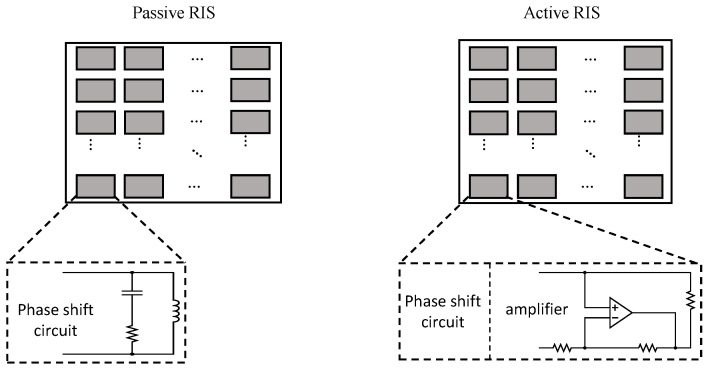
Passive RIS vs. active RIS.

However, in situations where both the direct and reflected links are blocked, a single RIS-aided communication system cannot be usable. Therefore, this paper considers a double RIS-aided communication system, as shown in Figure 1. The difference between the conventional system, as in Figure 2, and the proposed system, is as follows. In a single RIS system, the signal is reflected once by one RIS. However, in a proposed system, the signal that is reflected twice by two RISs is transmitted through three channels. Thus, obstacles between RIS 1 and the users do not interfere with communication. However, there is one serious problem in this system: The signal is affected by fading three times; thus, the signal is attenuated more than in the single RIS system. To solve this problem, the active RIS is considered in this paper. In [10,11,12], the active RIS is described as a rectangular surface similar to RIS, but its elements include an amplifier, as shown in Figure 3. The energy consumption of the active RIS is 6∼20 mW [13] and the energy consumption of the conventional RIS is 5 mW [7]. Thus, in order to maintain adequate system performance, the number of active RIS elements is important. For this reason, in this paper, a communication system assisted by a double RIS, composed of partially active elements, is proposed. In order to overcome too many obstacles and attenuation problems, this paper proposes a system that combines a double RIS system and an active RIS system.

### 1.1. Contributions

In [14], an active RIS was proposed to enhance spectrum efficiency and energy efficiency. In contrast to this paper, previous studies applied active RIS and single RIS systems. A double RIS system was proposed in [15]. Furthermore, in [16], a new RIS architecture, named portion active, was proposed to optimize the system capacity. The main contributions are summarized as follows:

- First, a double RIS system composed of partially active elements in MU-MISO is proposed. A double RIS system can overcome obstacles, while a RIS composed of active elements can mitigate triple fading effects. By using these schemes, communication performance can be enhanced in situations where both the direct and reflected links are blocked.
- Second, the sums of the covariance matrix and singular value decomposition are exploited to optimize matrix $\Theta_{1}$ of the RIS 1 elements. A similar method is used to optimize matrix $\Theta_{2}$ of the RIS 2 elements.
- Finally, the singular value of all channels is exploited to overcome substantial attenuations. If the singular value is less than the threshold, then the amplifier is activated; conversely, it is not activated.

### 1.2. Notation

Vectors and matrices are denoted in bold for lower and upper letters, respectively. $a_{i}$ and $∥\begin{array}{c} a \end{array}∥$ refer to the *i*-th element and L2-norm of vector a, respectively. $A^{H}$ and ${∥\begin{array}{c} A \end{array}∥}_{F}$ refer to the conjugate transpose and Frobenius norm of matrix A, respectively. R and C denote the real and complex number sets, respectively. $\mathrm{diag}\left(\begin{array}{c} a_{1},\cdots ,a_{N} \end{array}\right)$ refers to a diagonal matrix with diagonal elements with $a_{1},\cdots ,a_{N}$. exp(A) and ∠(A) are exponential of *A* and the angle of *A*.

### 1.3. Organization

Section 2 presents the communication system assisted by a double RIS composed of partially active elements. Section 3 proposes a scheme that exploits singular value decomposition to optimize the RIS reflective element and determine the amplifying elements. Section 4 presents the simulation results of the proposed RIS system and conventional RIS system. The conclusions are presented in Section 5.

## 2. System Model

This paper considers a double RIS-assisted multi-user multi-input single-output (MU-MISO) system; see Figure 1. The base station (BS) with *M* antennas communicates with the *u*-th user, who has a single antenna. The direct link between the base station (BS) and users is obstructed by various obstacles; hence, the direct link can be ignored. Also, the link between RIS 1 and users is blocked by many obstacles; hence, this link can also be ignored. The total number of RIS reflective elements and the total number of horizontal and vertical reflective elements are *N*, $N_{1}$, and $N_{2}$ with $N/N_{1}=N_{2}$. The active RIS is applied to RIS 2. We assume that perfect channel state information of the system is available at the BS.

Let $H_{BS}\in C^{N\times M}$ be the BS to the RIS 1 channel, $H_{inter}\in C^{N\times N}$ be the RIS 1 to RIS 2 channel, and $H_{user}\in C^{U\times N}$ be the RIS 2 to the user’s channel, with U=[1,···,U]. $\theta_{l}=\left[\theta_{1},\cdot \cdot \cdot ,\theta_{N_{l}}\right]=\left[\beta_{1}e^{\phi_{1}},\cdot \cdot \cdot ,\beta_{N_{l}}e^{\phi_{N_{l}}}\right]\in C^{N_{l}\times 1}$ denotes the reflection coefficient of RIS *l*, where β∈[0,1], ϕ∈[0,2π] and l=[1,2] are the amplitude, phase of the reflective element at the RIS *l*, and the index of the RIS, respectively. The received signal at the *u*-th user through the double reflective channel is given as follows:

(1) $$y_{u}=H_{user,u}\Theta_{2}H_{inter}\Theta_{1}H_{BS}x+n\in C^{U\times M},$$

where $\Theta_{l}=\mathrm{diag}\left(\theta_{l}\right)\in C^{N_{l}\times N_{l}}$ and $n\in C^{U\times M}$ denote the diagonal matrix of the RIS *l* and the addictive white Gaussian noise (AWGN) matrix with independent and identically distributed (i.i.d.) entries of zero mean and variance $\sigma^{2}$. x is a pilot symbol, which is simply set as x=1. The received signal can then be expressed as follows:

(2) $$y_{u}=H_{user,u}\Theta_{2}H_{inter}\Theta_{1}H_{BS}+n\in C^{U\times M}.$$

Then, the signal-to-interference-plus-noise-ratio (SINR) of this system at the *u*-th user is given as follows:

(3) $$\gamma_{u}=\frac{{∥H_{eff,u}w_{u}∥}^{2}}{\sum_{i=1,i\neq u}^{U}{∥H_{eff,u}w_{i}∥}^{2}+{∥eH_{active}∥}^{2}\sigma_{a}^{2}+\sigma^{2}},$$

where e and $\sigma_{a}^{2}$ are vectors indicating the active element and noise power caused by the amplifier. $H_{eff,u}$ and $H_{active}$ denote $H_{user}\Theta_{2}H_{inter}\Theta_{1}H_{BS}$ and $H_{user}\Theta_{2}$. Moreover, $w_{u}=H_{eff,u}{\left(H_{eff,u}H_{eff,u}^{H}\right)}^{-1}$ is the zero-forcing (ZF) transmit beamforming matrix at the *u*-th user. The ZF beamforming matrix should be normalized by factor $w_{factor}$, as follows:

(4) $$w_{factor}=\frac{\sqrt{p}}{{∥W∥}_{F}},$$

where *p* denotes the transmit power.

## 3. Proposed Scheme

In this paper, we propose a communication system assisted by a double RIS composed of partially active elements. First, matrix $\Theta_{1}$ of the RIS 1 elements is optimized by exploiting $H_{inter}$ and $H_{BS}$, which are related to the phase of $\Theta_{1}$. Then, the sum of the covariance matrix of $\mathrm{diag}\left(H_{inter}\right)H_{BS}$ for each element of RIS 1 is found, and the right singular value by using singular value decomposition (SVD) is exploited to the covariance matrix.

(5) $$T_{1}=svd\left(\sum_{n=1}^{N}H_{n}{\left(H_{n}\right)}^{H}\right)=U_{1}\sum_{1}V_{1}^{H},$$

where $H_{n}$ denotes $\mathrm{diag}\left(H_{inter,n}\right)H_{BS}$. The phase of matrix $\Theta_{1}$ of the RIS 1 elements can be optimized by using the right singular value $V_{1}$ obtained from Equation (5), as follows:

(6) $$\Theta_{1}=\mathrm{diag}\left(exp\left(j∠\left(V_{1}\right)\right)\right).$$

This optimization scheme does not randomly determine the angle of the RIS elements, but uses the right singular vector, including the channel information, to determine the angle of the RIS elements.

Obtaining the optimized phase of RIS 2 follows a similar method as described above. By considering the channel between the BS and RIS 2 as one channel, the received signal y can be expressed as follows:

(7) $$y_{u}=H_{user,u}\Theta_{2}H_{1},$$

where $H_{1}=H_{inter}\Theta_{1}H_{BS}$ denotes the channel between the BS and RIS 2. Similar to $\Theta_{1}$, the covariance matrix of $\mathrm{diag}\left(H_{user,u}\right)H_{1}$ is found and the right singular value by using SVD is exploited to the covariance matrix as follows:

(8) $$T_{2}=svd\left(\sum_{u=1}^{U}G_{u}{\left(G_{u}\right)}^{H}\right)=U_{2}\sum_{2}V_{2}^{H},$$

where $G_{u}$ denotes $\mathrm{diag}\left(H_{user,u}\right)H_{1}$. The phase of matrix $\Theta_{2}$ of RIS 2 elements can be optimized by using the right singular value $V_{2}$ obtained from Equation (8).

(9) $$\Theta_{2}\Theta =\mathrm{diag}\left(exp\left(j∠\left(V_{2}\right)\right)\right)$$

The singular value of the sum of covariance values is checked to determine which element’s amplifier to activate. If the singular value is less than the threshold, the amplifier will turn on, and vice versa. This is equivalent to Algorithm 1.
**Algorithm 1** Scheme to calculate amplifier vector m1:**for** n=1:N **do**2:    **if** $\sum_{2}<threshold$ **then**3:        $m_{n}=\eta$4:    **else**5:        **if** $\sum_{2}>=threshold$ **then**6:           $m_{n}=1$7:        **end if**8:    **end if**9:**end for**

Therefore, the amplitude and phase of matrix $\Theta_{2}$ of RIS 2 elements can be expressed as follows:

(10) $$\Theta_{2}=diag\left(mexp\left(j∠\left(V_{2}\right)\right)\right),$$

where $m\in R^{1\times N}$ denotes the active mode vector of each element. When the amplifier is turned on, the value of m is η, and when the amplifier is turned off, the value of m is 1.

## 4. Simulation Results

This paper considers the MU-MISO communication system assisted by the double RIS with M=64, U=6, N=64 and $N_{1}=8$. Moreover, BS, RIS 1, RIS 2, and the user are located at (1 m, 0), (0, 0.5 m), (0, 99.5 m), and (1 m, 100 m), respectively. Every channel in this system is subject to small-scale and large-scale fading. The large-scale fading follows a distance-based path-loss model, as $\mathrm{PL}\left(d_{x}\right)=c{\left(d_{x}/d_{0}\right)}^{-n}$, where *c*, $d_{x}$, and *n* represent the path-loss exponent at a reference distance $d_{0}$ and the distance between each node and the path-loss exponent. All three channels have a line-of-sight (LoS) path. Therefore, all channels experience small-scale fading that follows a Rician fading. Rician fading channel coefficients can be represented as follows:

(11) $$H=\sqrt{\mathrm{PL}(d_{x})}\left(\sqrt{\frac{K}{1+K}}H_{LoS}+\sqrt{\frac{1}{1+K}}H_{NLoS}\right),$$

where *K*, $H_{LoS}$, and $H_{NLoS}$ represent the Rician factor, LoS channel, and NLoS channel, respectively. The NLoS channel follows a Rayleigh distribution with zero mean and unit variance. The remaining parameters are listed in Table 1.

The sum rate *R* of this paper can be expressed as follows:

(12) $$R=\sum_{u=1}^{U}log_{2}\left(1+\gamma_{u}\right).$$

**Figure 4:**
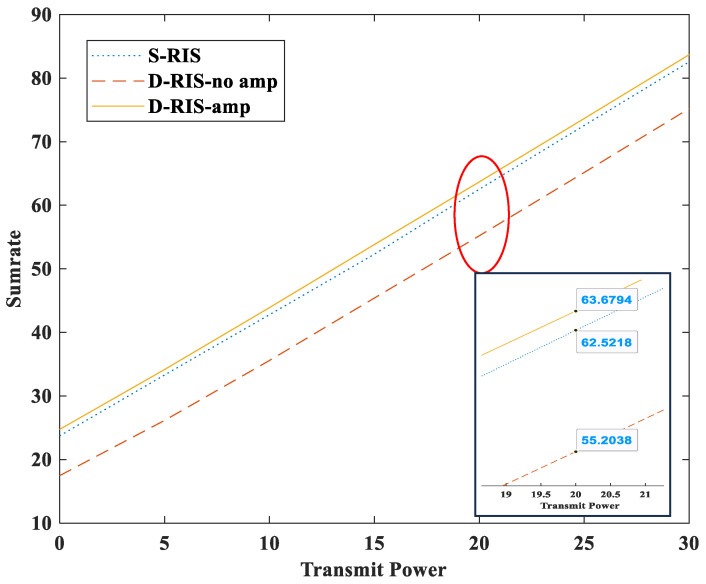
Sumrate versus transmit power (dBm) for comparison of existing and proposed schemes.

In Figure 4, S-RIS refers to the single RIS communication system, while D-RIS-amp and D-RIS-no amp refer to the double RIS communication system with a partial amplifier and without an amplifier. This simulation result demonstrates that D-RIS-amp outperforms the S-RIS in terms of the sum rate. However, due to the presence of numerous obstacles, the S-RIS is deemed infeasible. Therefore, the proposed RIS system will be useful in urban areas with many buildings. Moreover, the performance of the D-RIS-no amp has low performance compared to other schemes. This is because the double RIS system is affected by fading three times; hence, it cannot deliver sufficient performance unless an amplifier is used.

**Figure 5:**
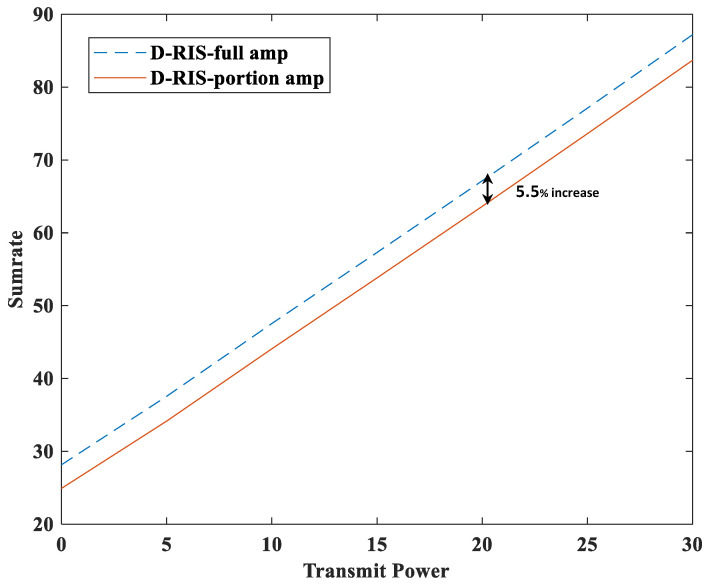
Sumrate versus transmit power (dBm) for comparison of full and partial amplifier.

**Figure 6:**
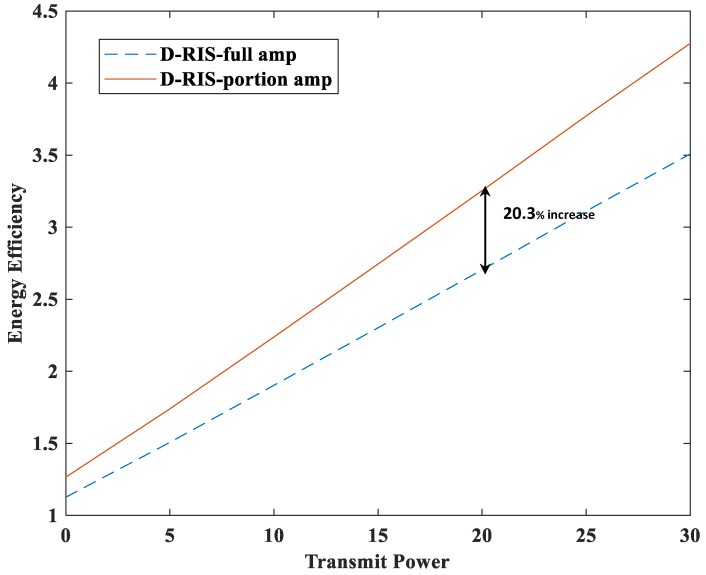
Energy efficiency versus transmit power (dBm).

Figure 5 and Figure 6 show the sum rate and energy efficiency comparison between the double RIS system with all amplifiers and the double RIS system with partial amplifier. In Figure 5 and Figure 6, D-RIS-full amp denotes the double RIS system in which the amplifiers of all elements are used when the RIS elements are amplified. The total power consumption of this system can be expressed as follows [17,18]:

(13) $$P_{total}=\frac{1}{\tau }\sum_{u=1}^{U}{∥w_{u}∥}^{2}+MP_{BS}+2NP_{ele}+\frac{1}{\tau }P_{A},$$

where τ=0.45 denotes the power amplifier efficiency, $P_{BS}=100$ W denotes the power consumption of one BS antenna, and $P_{ele}=2$ mW denotes the power consumption of one RIS element. $P_{A}$ is the amplification power of the active RIS element and it can be expressed as follows:

(14) $$P_{A}=\sum_{u=1}^{U}{∥\Theta_{2}H_{1}w_{u}∥}^{2}+\sigma_{a}^{2}{∥\Theta_{2}∥}^{2}.$$

The energy efficiency of this system can be expressed as follows:

(15) $$\eta ={\frac{R}{P}}_{total}.$$

Figure 5 shows that the D-RIS-full amp has a 5.5% better sum rate performance than the proposed RIS because the D-RIS-full amp amplifies all elements of the RIS, while the D-RIS partial amp amplifies a part of the RIS elements. However, Figure 6 shows that the D-RIS partial amp system has a 20.3% better performance in terms of energy efficiency than the D-RIS-full amp system. The trade-off between the two systems is the sum rate and energy efficiency. Although the sum rate performance is reduced by 5.5%, it is improved by 20.3% in terms of energy efficiency, so it seems that it will be better to use the proposed RIS system in the future when the efficient use of energy becomes important.

## 5. Conclusions

In this study, we propose a double RIS with a partially active assisted MU-MISO communication system. The proposed scheme optimizes the RIS elements and selects active elements through SVD. The proposed scheme has low complexity and a shorter simulation time compared to other optimization schemes. However, it has lower performance compared to schemes with higher complexity. In the simulation results, the proposed system delivers better sum rate performance than a single RIS-assisted communication system. Moreover, the proposed system has less sum rate performance than the double RIS with a fully active elements-assisted communication system. However, the proposed system has better energy efficiency than a double RIS with a fully active elements-assisted communication system. As a city grows and the number of buildings increases, the proposed system will be effectively used in urban areas. Therefore, the use of the proposed system will become more attractive as energy efficiency becomes more important.

## Figures and Tables

**Table 1:** Simulation parameters.

Parameter	Value
Iteration number	10,000
c	−30 dB
path-loss exponent for $H_{inter}$	3
path-loss exponent for $H_{user}$, $H_{BS}$	2.2
noise power ($\sigma^{2}$)	−80 dBm
noise power of amplifier ($\sigma_{a}^{2}$)	−76 dBm
amplifier power (η)	2
transmit power	30:5:50 dB
Rician factor (K)	10

## Data Availability

Not applicable.
